# The road to long-term memory: Top-down attention is more effective than bottom-up attention for forming long-term memories

**DOI:** 10.3758/s13423-020-01856-y

**Published:** 2021-01-14

**Authors:** Edyta Sasin, Daryl Fougnie

**Affiliations:** Department of Psychology, New York University Abu Dhabi, Abu Dhabi, United Arab Emirates

**Keywords:** Top-down attention, Bottom-up attention, Attentional capture, Long-term memory

## Abstract

Does the strength of representations in long-term memory (LTM) depend on which type of attention is engaged? We tested participants’ memory for objects seen during visual search. We compared implicit memory for two types of objects—related-context nontargets that grabbed attention because they matched the target defining feature (i.e., color; top-down attention) and salient distractors that captured attention only because they were perceptually distracting (bottom-up attention). In Experiment 1, the salient distractor flickered, while in Experiment 2, the luminance of the salient distractor was alternated. Critically, salient and related-context nontargets produced equivalent attentional capture, yet related-context nontargets were remembered far better than salient distractors (and salient distractors were not remembered better than unrelated distractors). These results suggest that LTM depends not only on the amount of attention but also on the type of attention. Specifically, top-down attention is more effective in promoting the formation of memory traces than bottom-up attention.

**Supplementary Information:**

The online version contains supplementary material available at 10.3758/s13423-020-01856-y.

We encounter millions of objects every day. While our ability to retain some of these objects in visual long-term memory (VLTM; a large-capacity, passive storage system for visual episodic memories) is surprisingly high and detailed (Brady, Konkle, Alvarez, & Oliva, 2008; Konkle, Brady, Alvarez, & Oliva, 2010; Shepard, 1967; Standing, Conezio, & Haber, 1970; Vogt & Magnussen, 2007), many of these objects are either not encoded or forgotten from memory (Lew, Pashler, & Vul, 2016; Mercer & Jones, 2019). What factors determine whether an item will or will not be stored in memory? On the one hand, previous research has identified many factors that influence whether something will be encoded in long-term memory (LTM). For example, stimuli are more likely to be remembered if they are repeated (Williams, 2010b), are processed deeply (Craik & Lockhart, 1972), carry emotional or personal importance (Hamann, 2001; Kensinger, Garoff-Eaton, & Schacter, 2007; Loftus, Loftus, & Messo, 1987), appear together with a target of another task (attentional boost effect; Swallow & Jiang, 2010, 2013), or are salient (Celikkale, Erdem, & Erdem, 2015). On the other hand, attentional lapses during encoding (deBettencourt, Norman, & Turk-Browne, 2018) or demanding concurrent tasks (Evans & Baddeley, 2018) can lead to worse memory. However, there is little research exploring how important is visual attention for successful encoding into VLTM. In one study, it was found that targets of a visual search task showed better memory performance than distractors in a surprise recognition test, suggesting that the increased attention given to targets was important for successful memory encoding (Williams, Henderson, & Zacks, 2005). However, the influence of attention on VLTM is certainly more complex than that captured by these findings.

The paucity of work relating VLTM and attention is quite surprising, given the extensive work aimed towards understanding attention and WM. Visual attention is known to have a critical role for encoding into visual WM. For example, stimuli that appear outside of attention often go undetected, as illustrated by research on change detection (Hollingworth, 2004; Rensink, 2002; Simons & Rensink, 2005), the attentional blink (Raymond, Shapiro, & Arnell, 1992; Vogel, Luck, & Shapiro, 1998), or inattentional blindness (Nakayama, Deutsch, & Nakayama, 1999; Simons & Chabris, 1999).

The goal of the present work is to further understand the role of attention in successful VLTM performance. In particular, attention is not a unitary construct. There is clear behavioral and neural evidence for separate attentional systems for purposefully attending to something (top-down) versus attending to salient parts of the environment (bottom-up) (Awh, Belopolsky, & Theeuwes, 2012; Connor, Egeth, & Yantis, 2004; Corbetta & Shulman, 2002; Pinto, Leij, Sligte, Lamme, & Scholte, 2013; Theeuwes, 2010). Top-down attention is shifted voluntarily, according to the current goals of the observer. Bottom-up attention, on the other hand, is captured in a stimulus-driven manner, by stimuli that differ significantly from surrounding inputs (Awh et al., 2012; Corbetta & Shulman, 2002; Egeth & Yantis, 1997; Theeuwes, 2010).

Given that attention is composed of two (at least partially) distinct mechanisms, does the benefit of attention for memory depend on which form is engaged? Here we examined the implicit memory of objects presented during a visual search task while manipulating the type of attention. More specifically, we compared the implicit memory of two types of objects—related-context nontargets that grabbed attention because they matched a target feature (top-down attention) and salient distractors that captured attention only because they were perceptually distracting (bottom-up attention). Note that capture by an object that shares a feature with a target held in memory is operationalized as top-down capture rather than priming effect (i.e., the facilitation of the processing of a stimulus due to the prior presentation of a stimulus that is perceptually or semantically related; Kristjánsson & Campana, 2010). Such a distinction is also consistent with studies showing that recent exposure to an object is insufficient to elicit capture by matching distractors, and that only representations held in WM might guide attention. (Olivers, Meijer, & Theeuwes, 2006; Soto, Heinke, Humphreys, & Blanco, 2005; Soto, Humphreys, & Rotshtein, 2007).

The magnitude of attentional capture was estimated from reaction times in the search task. The amount of capture is typically used to infer the amount of attention allocated to the distractors (Folk & Remington, 2008; Olivers, 2009; Olivers et al., 2006; Posner, 1980; van Moorselaar, Battistoni, Theeuwes, & Olivers, 2015; Yantis & Hillstrom, 1994). It should be pointed out that the amount of attention might be a sum of two components of attentional capture: the time of attentional focus on an object and the number of shifts of attention towards the object. Better VLTM performance for one of these distractors would suggest that encoding into VLTM depends on the type of attention that is engaged.

## Experiment 1

### Method

#### Participants

According to pilot studies, in which we found an effect size of ${\eta}_p^2$ = .35, α = .05, and power = 0.95, the sample size of a minimum 17 was required to find a significant effect in the memory performance with 95% probability, if the effect exists. Seventeen students and staff of the New York University of Abu Dhabi (12 males; *M* = 26 years, *SD* = 7.27) participated in the experiment in exchange for course credit, or alternatively received a subsistence allowance of 50 AED per hour. All participants had normal or corrected-to-normal visual acuity and gave informed consent. The experiments were approved by the New York University Abu Dhabi Institutional Review Board.

#### Apparatus and stimuli

Stimuli were presented using Psychtoolbox for MATLAB (Brainard, 1997), and the experiments were run on computers that were fitted with a 22-inch BenQ XL2411 monitor (144 Hz refresh rate, 1,920 × 1,080 pixels). All stimuli were presented on a black background at a viewing distance of 57 cm. The stimulus set consisted of 540 pictures of categorically distinct objects pulled from the Brady, Konkle, Gill, Oliva, and Alvarez (2013) data set. Twenty-four of these pictures were used only in the practice block. Three hundred sixty pictures were used in a search task (90 as targets, 30 as salient distractors, 30 as related-context nontargets, and 210 as distractors), and 90 pictures were used only in the surprise memory test as the novel objects. The pictures were assigned to conditions randomly on a per participant basis. Each picture was fit to a square of 100 × 100 pixels (2.92° × 2.92°). Importantly, each image had an object defined by a single, dominant color (e.g., a *blue* couch). Color was used to define the search target. At the beginning of each trial, participants were given a cue—a centrally presented colored circle (radius 0.90° visual angle) to indicate the target color. The target colors were selected randomly from a set of four possible colors that were created by manipulating the color of the target image. Specifically, the color was the target image’s dominant color shifted by 0°, 90°, 180° or 270° (Brady et al., 2013) in hue space using the LAB circular color space. Critically, there were three types of trials in the search task. On *neutral* trials, the search display contained the target and three distractors. On *salient distractor* trials, the search display contained the target, a salient distractor, and two distractors. A salient distractor is defined as a distractor with a color unrelated to the target item, but which has increased bottom-up salience because it flickered on the screen (the other items were presented without flicker). The salient distractor flickered rapidly (appear and disappear) at frequency rates chosen randomly during a trial from the frequencies between 0.3 and 0.9 Hz. On *related-context nontarget trials*, the search display contained the target, related-context nontarget, and two distractors. A related-context nontarget is one whose color was similar to, but not exactly the same as, the target color (i.e., shifted by 30° in hue space from the hue of the target). Note that this distractor was related to the target by color, the defining feature of the search task, but the object identity and locations were completely independent. The colors of the unrelated distractors or salient distractor were chosen randomly from the set of four colors, excluding the target color (e.g., if the target image’s color was shifted by 90° in hue, other images could have colors shifted by 0°, 180°, or 270° in hue). Both salient distractors and related-context nontargets were never targets. Critically, the related-context nontarget and salient distractor conditions were found to be equally distracting in pilot experiments.

The search display was composed of the four different objects located equidistantly on an imaginary circle of radius 4.38° around fixation, with locations determined randomly on a per trial basis. The items were at 45°, 135°, 225°, and 315° positions. The boundaries of each object were separated by at least 1.46° of visual angle.

#### Procedure

**Fig. 1:**
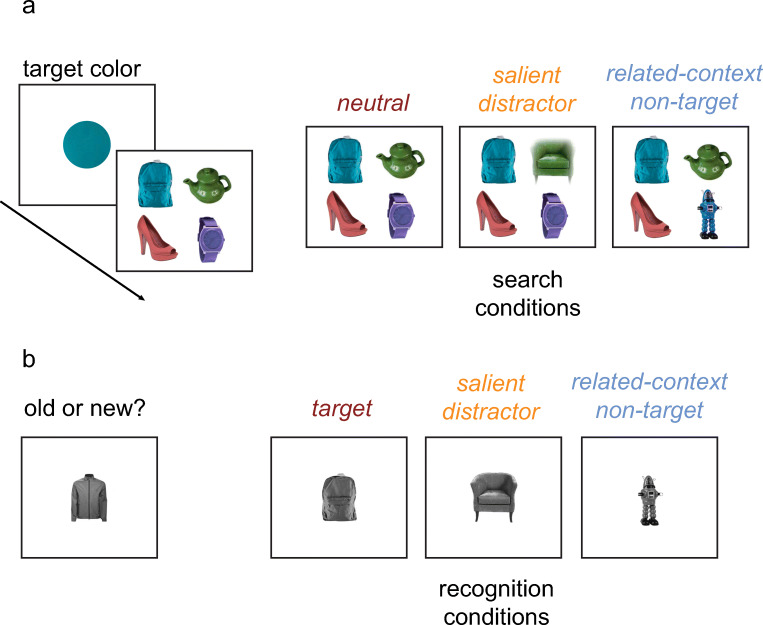
**a** The structure of the search task. Participants searched for an object of a specific color that changed on a per-trial basis. In *neutral* conditions, the search display contained the target and unrelated distractors. In *related-context nontarget* conditions, one of the distractors has a color similar to, but not the same as, the target color (in this example, it is a blue robot). In *salient distractor* conditions, one of the distractors flickered rapidly at a random frequency (in this example, it is a green armchair). **b** The structure of the recognition test. Participants were asked to indicate whether an object on the screen had been shown earlier on in the experiment. Memory was separately assessed for search targets, salient distractors, and related-context nontargets

The experimental procedure is illustrated in Fig. 1. At the beginning of each trial, participants were presented with a target color for 1,000 ms, which was followed by a 500-ms blank interval. Subsequently, the search display appeared on the screen. The task participants were given was to localize the object with a color matching the target color.

To respond, participants indicated the search target location by pressing one of the four keys (“A,” “K,” “Z,” “M”) that corresponded to the location on the screen. Participants were instructed to make the search responses as quickly as possible. The search display remained on the screen until a response was made or until the maximum presentation time of 2 s was reached. (only 0.4 % of displays reached the 2-s presentation time). Participants were required to make a response to continue to a new trial even on trials in which when the search display was removed after 2 s. All pictures (90 targets, 30 salient distractors, 30 related-context nontargets, and 210 as unrelated distractors, chosen randomly for each participant) were repeated four times during the search session. Thus, there were 120 trials for each search condition, resulting in 360 trials in total. The conditions were intermixed and were presented in random order. The experiment was preceded by six practice trials to give participants familiarity with the task. The experimental phase was followed by an unexpected recognition test that required participants to indicate whether an object on the screen had been shown earlier on in the experiment. The recognition test included 30 targets from the neutral search condition, 30 salient distractors, 30 related-context nontargets, and 90 novel objects. Importantly, the novel objects were not presented during the experiment. Participants were instructed to respond by pressing “Z” when an object was identified as “old” (this was considered correct for the target and distractors) and “M” when the object was identified as “new” (this was considered correct for novel objects). These objects were shown randomly, one at a time. The color of each object presented in the recognition test was converted to grayscale. Participants were told to respond accurately (speed was not stressed, and the object remained in view until the judgment was made).

### Results

**Fig. 2:**
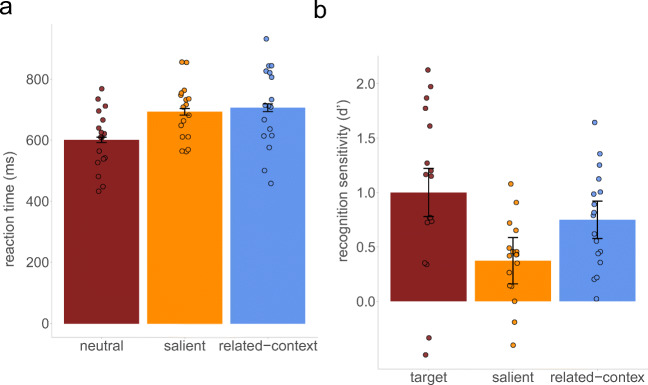
The results of Experiment 1. **a** Mean RTs (ms) in the search task as a function of the search condition. **b** Mean sensitivity index (*d'*) as a function of recognition conditions. Error bars reflect 95% within-subjects confidence intervals (Cousineau, 2005; Morey, 2008)

Correct search trials made up 88% of the data (91% in both the neutral condition, 92% in the salient distractor condition, and 82% in the related-context nontarget condition). However, due to human error, the accuracy of two trials from each condition could not be determined, which lead to an overall slightly lower accuracy than would be expected in this task. Before analyzing the reaction times (RTs) for the search task, we excluded the trials with incorrect responses in the search task. Next, we excluded trials with search-RTs shorter than 150 ms or longer than 3,000 ms and trials with search RTs above a cutoff value of three standard deviations from the mean. This procedure resulted in a loss of 2.59% of the data points. Importantly, none of the qualitative conclusions are altered by excluding the aforementioned trials. Search reaction times and sensitivity are illustrated in Fig. 2.

We performed a repeated-measures analysis of variance (ANOVA) on search RTs with search condition as a factor (*neutral* vs. *salient distractor* vs. *related-context nontarget*). This showed a significant effect of search type, *F*(2, 32) = 43.63, *p* < .001, ${\eta}_p^2$ = .73. Planned contrasts revealed that salient distractor trials (695 ms, 95% CI [684, 705]) resulted in slower RTs relative to neutral trials (602 ms, 95% CI [594, 611]), *t*(16) = 12.67, *p* < .001, *d* = 3.07. Similarly, related-context nontarget trials (708 ms, 95% CI [695, 721]) resulted in slower RTs relative to neutral trials, *t*(16) = 8.58, *p* < .001, *d* = 2.08. There was no significant difference between salient distractor trials and related-context nontarget trials, *t*(16) = 0.85, *p* = .406. Further, there is no evidence that capture by the salient distractor was initially strong and then had a reduced effect in later trials (see Supplemental Material).

The current results demonstrate not only that both types of distractors captured attention but also suggests that the amount of attention afforded to each distractor was equivalent for the two conditions. Given the evidence of *equal* attentional capture for both distractor conditions, we can examine performance on the recognition task as a function of the *type* of attention.

Performance on the surprise recognition task was also examined. To ensure that analysis of memory was done only on trials where participants successfully identified the target, the recognition test analyses were restricted to stimuli from trials with correct search responses. To measure the degree to which objects were encoded into memory, we calculated a sensitivity index (*d'*), a signal detection measure conceptualized as the distance between signal and noise distributions^1^ (Stanislaw & Todorov, 1999). A one-tailed one-sample *t* test was performed on sensitivity indices *d'* for each memory condition: targets, salient distractors, and related-context nontargets. The one-tailed *t*-test evaluations showed that in each memory condition, *d'* was significantly above zero (all *t*s > 4.13, all *p*s < .001), indicating that participants were able to remember something about the items even though they were not informed about the test in advance. Next, sensitivity indices *d'* were entered in an ANOVA, with memory condition as a factor. This analysis revealed significant effect of memory condition, *F*(2, 32) = 10.81, *p* < .001, ${\eta}_p^2$ = .40. A series of post hoc comparisons were then performed using the Holm–Bonferroni correction. These comparisons showed that the memory performance was better for the targets (*d'* = 1.00, 95% CI [0.78, 1.22]) than the salient distractors (*d'* = 0.37, 95% CI [0.16, 0.59]), *t*(16) = 4.08, *p* = .003, *d* = 0.99. The difference in memory performance between the targets and the related-context nontargets (*d'* = 0.75, 95% CI [0.58, 0.92]) did not quite reach significance, *t*(16) = 1.95, *p* = .07, *d* = 0.47. Importantly, the memory performance was better for the related-context nontargets than for the salient distractors, *t*(16) = 3.07, *p* = .015, *d* = 0.75, suggesting that the attentional capture by those distractors was more successful in encoding/storage into LTM.^2^

First, it is important to highlight that analysis of memory performance in Experiment 1 showed that search targets, related-context nontargets, and salient distractors were indeed encoded into LTM even if participants were not asked to memorize these objects. Such incidental encoding led to overall small sensitivity indices, which was expected, taking into account that executing the search task does not require any identification of the presented objects. Moreover, objects in the recognition test were grayscale versions of colored objects presented during the search task, which might also contribute to overall small sensitivity indices, according to encoding specificity (Tulving & Thomson, 1973). Crucially, the results showed the same magnitude of attentional capture produced by related-context nontargets (top-down capture) and salient distractors (bottom-up capture). However, the surprise recognition test revealed that the memory performance was much better for the related-context nontargets than the salient distractors.

Search targets were better remembered than the other stimuli (although the difference in memory between targets and related-context nontargets did not reach significance level, perhaps due to insufficient statistical power). This replicates results from previous studies (Tatler & Tatler, 2013; Williams, 2010a, 2010b; Williams et al., 2005) and suggests that objects that are encoded as targets are better encoded into LTM.

## Experiment 2

The critical finding of Experiment 1 is that salient distractors were remembered less than related-context nontargets, suggesting that bottom-up attention is less efficient as a means to VLTM encoding compared with top-down attention. A possible criticism of the current design is that our method of inducing bottom-up saliency involved flickering the item, which means that the item was on the screen for a reduced amount of time. The goal of Experiment 2 is to replicate the findings of Experiment 1 and to test whether the differences in memory performance that we observed between salient distractors and related-context nontargets could be due to the nature of the flicker rather than the difference in the type of attention that is engaged. To control for this possibility, in Experiment 2, we used a different form of increasing stimulus salience. Specifically, we alternated stimulus luminance of the salient distractor. An additional goal of Experiment 2 was to measure memory performance of the unrelated distractor items to provide a baseline of distractor memory to compare to salient distractor memory.

### Method

#### Participants

Seventeen students of the New York University of Abu Dhabi (eight males; *M* = 20.3 years, *SD* = 1.28) participated in the experiment in exchange for course credit or received a subsistence allowance of 50 AED per hour. All participants had normal or corrected-to-normal visual acuity and gave informed consent. The experiments were approved by the New York University Abu Dhabi Institutional Review Board.

#### Apparatus and stimuli

The stimuli were identical to the ones used in Experiment 1, except for the following changes. All stimuli were presented on a white background. The luminance of the salient distractor was changed during a trial at a random frequency between 0.3 and 0.9 Hz. The luminance of the salient distractor alternated between original luminance and increased luminance (specifically, the L value of the distractor color, in CIE LAB space was increased by 60). The recognition test additionally included 30 old or new judgments on images that were used as unrelated distractors. There was no increase in the number of “new” images in the recognition test.

### Results

**Fig. 3:**
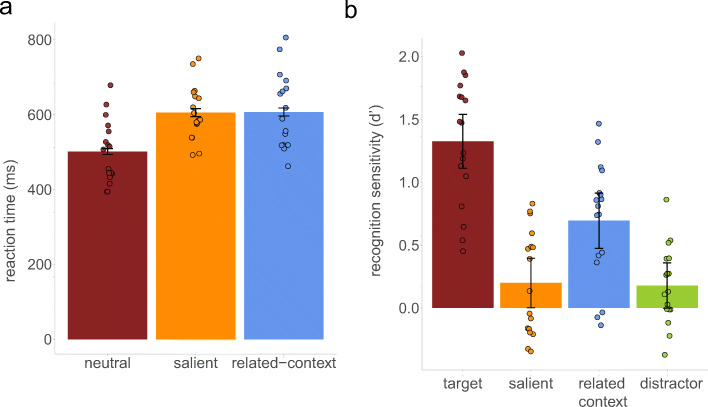
The results of Experiment 2. **a** Mean RTs (ms) in the search task as a function of the search condition. **b** Mean sensitivity index (*d'*) as a function of recognition conditions. Error bars reflect 95% within-subjects confidence intervals (Cousineau, 2005; Morey, 2008)

Participant’s performance on the search task was 93% (95% in both the neutral and the salient distractor conditions and 87% in the related-context nontarget condition). Before analyzing RTs for the search task, we excluded trials with incorrect search responses, RTs less than 150 ms or greater than 3,000 ms, or RTs greater than three standard deviations from that participant mean (resulting in a loss of 2.42% of data points). Importantly, the qualitative conclusions remain the same if the aforementioned trials are not excluded. An ANOVA was performed on search RTs, with search condition as a factor (*neutral* vs. *salient distractor* vs. *related-context nontarget*). The results, illustrated in Fig. 3, showed significant effect of search type, *F*(2, 32) = 45.20, *p* < .001, ${\eta}_p^2$ = .74. We found that RTs were longer for both the salient distractor condition (610 ms, 95% CI [599, 621]), *t*(16) = 9.98, *p* < .001, *d* = 2.42, and the related-context nontarget condition (612 ms, 95% CI [601, 23]), *t*(16) = 7.87, *p* < .001, *d* = 1.91, relative to the neutral condition (505 ms, 95% CI [497, 513]). There was no difference in RTs between the salient distractor condition and the related-context nontarget condition, *t*(16) = 0.11, *p* = .914. Again, the results demonstrate that there was no noticeable difference in the amount of captured attention between the distractors.

We also analyzed recognition test data, restricted to stimuli from trials where participants successfully identified the target. A one-tailed one-sample *t* test on sensitivity indices *d'* again showed that in each memory condition *d'* was significantly above zero (all *t*s > 1.96, all *p*s < .032), indicating that memory performances were significantly above chance, even for the unrelated distractors. The ANOVA on sensitivity indices *d'* further revealed significant effect of memory condition, *F*(3, 48) = 31.64, *p* < .001, ${\eta}_p^2$ = .66. The post hoc comparisons with Holm–Bonferroni correction showed that the targets (*d'* = 1.33, 95% CI [1.11, 1.54]) were remembered better than the salient distractors (*d'* = 0.20, 95% CI [0.00, 0.40]), *t*(16) = 8.62, *p* < .001, *d* = 2.09, the related-context nontargets (*d'* = 0.69, 95% CI [0.47, 0.91]), *t*(16) = 4.61, *p* = .001, *d* = 1.12, and unrelated distractors (*d'* = 0.18, 95% CI [0.00, 0.36]), *t*(14) = 7.68, *p* < .001, *d* = 1.86. There was not a significant difference in the memory performance between the salient distractors and unrelated distractors, *t*(16) = 0.19, *p* = .849. Most critically, we replicated the finding that the related-context nontargets were remembered better than the salient distractors, *t*(16) = 3.11, *p* = .013, *d* = 0.76. This again demonstrates that all forms of attention are not equal in terms of successful LTM performance.^3^

In Experiment 2, we replicated the pattern of the results showing that, despite the same costs in the search performance engendered by related-context nontargets and salient distractors, the latter one resulted in much weaker long-term memories. In fact, the memory for the salient distractors did not differ from the memory for other distractors.

## General discussion

The human sensory system is constantly bombarded by an enormous amount of information from the external world. Which pieces of this information will be retained in memory, and why? While this topic has received considerable focus, one area that is poorly understood is the role that visual attention plays in successful VLTM. To shed light on this question, the present work tested memory for distractors seen during a visual search task, while searching for a target object of a particular color. In one condition (related-context nontarget), we made a distractor item likely to receive top-down attention by making it have a feature (i.e., color) similar to the to-be-searched feature. In another condition (salient distractor), we introduced an item that was irrelevant to the search task, but that would attract bottom-up attention due to its stimulus salience. In Experiment 1, the distracting stimulus was flickering at a rapid rate during the search task. In Experiment 2, the flickering was replaced by alternations in luminance to generalize over distinct methods of introducing stimulus salience. As expected, the presentation of both the related-context nontarget and salient distractor led to slower search compared with the baseline condition, in which none of these distractors was presented. Additionally, the amount of distraction did not differ between the distraction conditions. Importantly, even though both distractors produced the same magnitude of attentional capture, the related-context nontargets were remembered better than the salient distractors, according to a surprise VLTM test administered at the end of the study. This provides the first direct evidence that the objects that grab top-down attention are more likely to be encoded in VLTM than the objects that grab bottom-up attention.

What is the mechanism by which purposeful attention leads to more effective encoding into VLTM? It is possible that different types of attention lead to the attention being paid to different features. Perhaps in the case of bottom-up capture, attention was more focused on the distracting property itself (e.g., flicker on Experiment 1) rather than on the identity of the salient object. Indeed, recent studies have shown that when attention is focused on the task-relevant attribute of the object, the other attribute of this object is not necessarily consolidated into memory (Chen, Swan, & Wyble, 2016; Chen & Wyble, 2015). Similarly, it is likely that attention was more focused on color when the related-context nontarget was presented. But perhaps when attention was focused on a salient feature, there is less attention left over to process other image attributes (e.g., identity) then when attention was being focused by color. Importantly, it should be noted that the task-relevant feature, color, was equally irrelevant for the LTM test (which was conducted on grayscale images) as the saliency manipulations.

Another possible explanation is that purposeful attention is useful for VLTM encoding because attending is more effortful when it occurs via top-down means than when it occurs due to bottom-up salience. One of the most influential findings in LTM research is the levels of processing effect (Craik & Lockhart, 1972; Schulman, 1971), which demonstrates that effortful processing of to-be-remembered items leads to better LTM encoding than shallow processing. This framework defines “effortful” as requiring semantic processing. Although our search task did not require any conceptual assessment, it is possible that the attention grabbed by distractors that share similar traits with the target, while equal with salient distractors in terms of negative effects on the search task, leads to something akin to more “effortful” processing.

The work also has practical relevance, particularly for advertisers and anyone interested in creating a lasting impression on the human mind. It appears that the effects of salience, while strong, are short-lived and do not lead to strong encoding in VLTM. In contrast, when attention is purposefully shifted towards information, it is retained longer, even in cases where there is no explicit requirement to memorize that information. Perhaps this is good news for those of us who are sick of flashing and distracting images on the television or websites. This strategy may be useful for catching our attention in the moment, but may not ultimately be an effective advertising technique if capturing our attention in this way does not lead to encoding into VLTM. Indeed, there is evidence showing that animated advertisements attract less attention than static ones (Lee & Ahn, 2012).

In conclusion, the current study suggests that the formation of visual long-term memories not only depends on the amount of attention but also the type of attention that is engaged. Specifically, even though the magnitude of bottom-up capture by salient distractors was the same as top-down capture by related-context nontargets, the related-context nontargets were remembered better than the salient distractors. While future studies are needed to clarify how exactly attention is distributed between targets and different type of distractors, the present data provide evidence that the road to long-term memory may take many paths, but that purposeful attention provides the quickest route.

## Supplementary Information

ESM 1 (DOCX 22 kb)

## Data Availability

The data for all experiments are available (https://osf.io/qyn5a/).

## Footnotes

1 Specifically, *d'* was calculated as a difference between *z*-transforms of hit rate (i.e. the proportion of “old” trials to which subject responded “old”) and false alarm rate (i.e., the proportion of “new” trials to which subject responded “old”), where *z*-transforms are inverse of the normal distribution cumulative density function. Note that the false-alarm rate for each participant was calculated based on incorrect responses to all “new” objects, and thus it was the same for all conditions.

2 Note that this analysis assumes that differences in hit rate arose because of differences in memory signal, not decision criterion, which is reasonable given the intermixed nature of our design. However, to further rule out differences in decision criterion, we conducted an additional analysis that fixed criterion across all memory conditions (see Supplemental Material).

3 We again conducted an additional analysis that fixed criterion across all memory conditions (see Supplemental Material), to ensure that our findings are not affected by differences in bias.
