# Supplementary material for: The road to long-term memory: Top-down attention is more effective than bottom-up attention for forming long-term memories
Source: Psychon Bull Rev. 2021 Jan 14;28(3):937–45. doi: 10.3758/s13423-020-01856-y (PMC8219582; doi:10.3758/s13423-020-01856-y)
Supplement: Supplementary file 1 — (DOCX 22 kb) [file 13423_2020_1856_MOESM1_ESM.docx]

**Supplemental Material**

**Search reaction time analysis of capture by salient distractor**

**Experiment 1.** The lack of difference between the salient and related-context non-targets in the search performance does not exclude the possibility that salient distractor produced much bigger capture across initial trials and smaller or no capture later in the task. Participants could quickly learn that salient distractor was always irrelevant, and thus they could try to avoid attending to it. To test this possibility, we examined the first and last 20% of the trials in regard to the attentional capture effect of the salient distractors. The difference in RTs between neutral (M = 621 ms, 95% CI [602, 639]) and salient distractor (M =725ms, 95% CI [702, 748) conditions computed for first 20% of trials was significant, *t*(16) = 4.98, *p* < .001, *d* = 1.21. The difference in RTs between neutral (M = 590 ms, 95% CI [572, 609]) and salient distractor (M = 675 ms, 95% CI [652, 699]) conditions was also significant for last 20 % of trials, *t*(16) = 8.49, *p* < .001, *d* = 1.21. There was no significant difference between magnitude of capture effects observed in first (M = 104 ms) and last (M = 85 ms) 20% of trials, *t*(16) = 0.94, *p* = .363, *d* = 0.23.

**Experiment 2.** We examined whether salient distractors produced the same magnitude of capture in the first and last 20 % of the trials. The difference in RTs between neutral (M = 524 ms, 95% CI [505, 544]) and salient distractor (M = 620 ms, 95% CI [596, 644) conditions computed for first 20% of trials was significant, *t*(16) = 5.91, *p* < .001, *d* = 1.43. The difference in RTs between neutral (M = 485 ms, 95% CI [472, 500]) and salient distractor (M = 605 ms, 95% CI [582, 630]) conditions was also significant for last 20 % of trials, *t*(16) = 6.77, *p* < .001, *d* = 1.64. There was no significant difference between magnitude of capture effects observed in first (M = 96 ms) and last (M = 119 ms) 20% of trials, *t*(16) = 1.03, *p* = .318, *d* = 0.25.

**D prime analysis controlling for decision criterion**

**Experiment 1.** We conducted an additional analysis of the recognition performance that assumed a shared decision criterion for all memory conditions, to ensure our findings are not affected by bias across conditions. Specifically, we calculated the mean criterion across conditions and participants and used this as fixed criterion to derive a condition specific false alarm rate from equitation for criterion measure $c=\frac{1}{2}(z\left( H \right)+z\left( F \right))$ (Macmillan & Creelman, 1991). We then computed d’ for each subject and each condition using derived false alarm rates. These sensitivity indices were entered in an ANOVA with memory condition as a factor. We found a significant effect of memory condition, *F*(2, 32) = 10.80, *p* < .001, $\eta_{p}^{2}$= .40. Post-hoc comparisons (Holm–Bonferroni correction) revealed that memory performance was better for targets (d’ = 1.29, 95% CI [0.85, 1.74]) than salient distractors (d’ = 0.38, 95% CI [-0.39, 0.46]), *t*(16) = 4.07, *p* = .003, *d* = 0.98, but the recognition of the targets was not different from related-context non-targets, *t*(16) = 1.94, *p* = .070, *d* = 0.47. Critically, memory performance for the related-context non-targets (d’ = 0.79, 95% CI [0.45, 1.13]) was better than for the salient distractors, (d’ = 0.38, 95% CI [-0.39, 0.46]]), *t*(16) = 2.81, *p* = .015, *d* = 0.75. Thus, this modified signal detection analysis found equivalent results, ruling out changes in bias as driving the findings.

**Experiment 2.** We again conducted additional analysis in which a shared decision criterion was assumed for all memory conditions. The ANOVA on these sensitivity indices d’ revealed significant effect of memory condition, *F*(3, 48) = 31.65, *p* < .001, $\eta_{p}^{2}$ = .66. The post hoc comparisons (Holm–Bonferroni correction) found that targets (d’ = 2.05, 95% CI [1.62, 2.48]) were remembered better than: salient distractors (d’ = -0.20, 95% CI [-0.60, 0.19]), *t*(16) = 8.62, *p* < .001, *d* = 2.09, related-context non-targets (d’ = 0.79, 95% CI [0.35, 1.23]), *t*(16) = 4.60, *p* = .001, *d* = 1.12 and unrelated distractors (d’ = -0.24, 95% CI [-0.60, 0.12]), *t*(14) = 7.67, *p* < .001, *d* = 1.86. There was no significant difference in the memory performance between the salient distractors (d’ = -0.20, 95% CI [-0.60, 0.19]), and unrelated distractors (d’ = -0.24, 95% CI [-0.60, 0.12]), *t*(16) = 0.19, *p* = .849. Critically, the related-context non-targets (d’ = 0.79, 95% CI [0.35, 1.23]) were remembered better than the salient distractors (d’ = -0.20, 95% CI [-0.60, 0.19]), *t*(16) = 3.12, *p* = .013, *d* = 0.75. Therefore, the two signal detection analyses were equivalent demonstrating that the results are unlikely influenced by response bias.

Macmillan, N. A., & Creelman, C. D. (1991). *Detection theory : a user’s guide*. Cambridge University Press.
